# Genomic Insights into Methicillin-Resistant Staphylococci and Mammaliicocci from Bulk Tank Milk of Dairy Farms in Serbia

**DOI:** 10.3390/antibiotics12101529

**Published:** 2023-10-11

**Authors:** Andrea Kos, Bojan Papić, Majda Golob, Jana Avberšek, Darja Kušar, Tijana Ledina, Jasna Đorđević, Snežana Bulajić

**Affiliations:** 1 Directorate for National Reference Laboratories, Ministry of Agriculture, Forestry and Water Management, Batajnički drum 7, 11186 Belgrade, Serbia; andrea.kos@minpolj.gov.rs; 2 Institute of Microbiology and Parasitology, Veterinary Faculty, University of Ljubljana, Gerbičeva 60, 1000 Ljubljana, Slovenia; bojan.papic@vf.uni-lj.si (B.P.); majda.golob@vf.uni-lj.si (M.G.); jana.avbersek@vf.uni-lj.si (J.A.); darja.kusar@vf.uni-lj.si (D.K.); 3 Department of Food Hygiene and Technology, Faculty of Veterinary Medicine, University of Belgrade, Bulevar Oslobođenja 18, 11000 Belgrade, Serbia; tijana.ledina@vet.bg.ac.rs (T.L.); jasna.djordjevic@vet.bg.ac.rs (J.Đ.)

**Keywords:** methicillin-resistant staphylococci/mammaliicocci, methicillin-resistant *Staphylococcus aureus*, bulk tank milk, antimicrobial resistance, virulence factors, whole-genome sequencing

## Abstract

The potential risk to human and animal health provides a rationale for research on methicillin-resistant staphylococci (MRS) and mammaliicocci (MRM) in dairy herds. Here, we aimed to estimate their occurrence in the bulk tank milk (BTM) samples collected in 2019–2021 from 283 bovine dairy farms in the Belgrade district. We used whole-genome sequencing to characterize the obtained isolates and assess their genetic relatedness. A total of 70 MRS/MRM were recovered, most frequently *Staphylococcus haemolyticus* and *Mammaliicoccus sciuri*. Five clusters of 2–4 genetically related isolates were identified and epidemiological data indicated transmission through, e.g., farm visits by personnel or milk collection trucks. Most MRSA isolates belonged to the typical livestock-associated lineage ST398-t034. One MRSA isolate (ST152-t355) harbored the PVL-encoding genes. Since MRS/MRM isolates obtained in this study frequently harbored genes conferring multidrug resistance (MDR), this argues for their role as reservoirs for the spread of antimicrobial resistance genes. The pipeline milking system and total bacterial count >100,000 CFU/mL were significantly associated with higher occurrences of MRS/MRM. Our study confirms that BTM can be a zoonotic source of MRS, including MDR strains. This highlights the urgent need for good agricultural practices and the continuous monitoring of MRS/MRM in dairy farms.

## 1. Introduction

*Staphylococcus aureus* is a well-known pathogen of many animal species and humans, and is associated with a variety of manifestations ranging from benign skin infections to life-threatening conditions [1]. In dairy herds, mastitis caused by *S. aureus* remains a major disease burden despite numerous herd management programs [2]. Although many different *S. aureus* genotypes circulate in dairy herds worldwide, a limited number of clones are responsible for most *S. aureus* mastitis cases, with clonal complexes (CCs) 1, 5, 8 and 97 being the most prevalent [2]. From a food safety perspective, *S. aureus* is considered one of the most important causative agents of food intoxication worldwide [3]. Conversely, coagulase-negative staphylococci, now more often referred to as non-*S*. *aureus* staphylococci (NAS) [4], have traditionally been considered non-pathogenic due to their established commensal relationship with humans and animals, although the pathogenicity of different NAS species and their involvement in bovine mastitis remain to be fully evaluated [4].

Methicillin-resistant staphylococci (MRS) have been repeatedly isolated from dairy herds, albeit at low prevalence rates [5]. A meta-analysis of published data showed that the estimated global prevalence of methicillin-resistant *S. aureus* (MRSA) from various sources on dairy cattle farms was 3.2% [6]. A slightly higher global prevalence of MRSA (4.3%) was reported for clinical and subclinical bovine mastitis cases [7]. Methicillin-resistant NAS, which are often isolated from dairy farms, serve as a potential reservoir of antimicrobial resistance (AMR) and virulence genes for mastitis-associated pathogens [8,9,10]. Since AMR genes are often located on mobile genetic elements, they can be horizontally transferred between different species of the family *Staphylococcaceae*, and the resistant mammaliicocci can also play a major role as AMR reservoirs for pathogenic staphylococci [4,11,12]. Namely, a novel genus *Mammaliicoccus* with five species (*M. sciuri*, *M. fleurettii*, *M. lentus*, *M. vitulinus* and *M. stepanovicii*) was recently reclassified from the genus *Staphylococcus* to comprise members of the former *Staphylococcus sciuri* group [13], and was reported to harbor AMR genes encoding resistance to several antimicrobial classes [12,14] and unusual SCC*mec* elements [12,15,16]. The presence of mammaliicocci on dairy farms has also been confirmed [10,12]. 

Agriculture has a long tradition in Serbia and accounts for 6.3% of the gross domestic product [17]. According to the official statistics for 2021, there are a total of 408,000 dairy cows in Serbia [17]. Milk production reaches 1473 × 10^6^ L/year, with an average milk yield of 3626 L/cow, and is heavily dependent on small, family-run farms. Despite the great importance of milk production in Serbia and the existence of a national mastitis control program under the national Regulation on Animal Health Program [18], its implementation in practice has not been successful due to lack of resources and defined protocols. In addition, reliable data on the farm use of antimicrobials are lacking [19]. Moreover, data on the prevalence of major mastitis-associated pathogens, average somatic cell counts (SCC) and total bacterial counts (TBC) in the bulk tank milk (BTM), are generally not available either at national or regional level. 

Studies on MRSA and other staphylococci from Serbia are scarce and mainly focused on the prevalence and characterization of MRSA from humans [20,21,22,23], and to a lesser extent on MRSA from farmed [24,25,26] and companion [27] animals. Since the first reported case of MRSA-associated subclinical bovine mastitis [26], efforts have been made to characterize dairy-related MRSA [28,29], but specific data on the prevalence and molecular epidemiology of MRS in the Serbian dairy supply chain are still missing.

Several studies used BTM samples to determine MRSA prevalence in dairy herds [30,31,32,33,34,35]. The objectives of the present study were to (i) estimate the occurrence of MRS and methicillin-resistant mammaliicocci (MRM) in BTM samples, (ii) perform an in-depth genetic characterization of the obtained MRS and MRM isolates using whole-genome sequencing (WGS) and (iii) identify risk factors associated with the occurrence of MRS/MRM in BTM. 

## 2. Results

### 2.1. The Occurrence of MRS/MRM in BTM Samples

A total of 283 BTM samples were collected from a different dairy farm each to evaluate the occurrence of MRS/MRM. The results showed that 68/283 (24.0%) farms were positive for MRS and/or MRM, with a total of 70 MRS/MRM isolates identified (Tables S1 and S2). Of the 68 farms positive for MRS and/or MRM, 66 were positive for either MRS or MRM. In addition, the farm with code 255 was positive for two different MRS species (*S. aureus* isolate MRSA4 and *S. haemolyticus* isolate MRS7), and the farm with code 250 was positive for MRS and MRM species (*M. fleurettii* isolate MRS36a_Sf and *S. haemolyticus* isolate MRS36_Sh) (Tables S1 and S2). In total, 41/283 (14.5%) farms were positive for MRS and 28/283 (9.9%) farms for MRM; the percentage of MRSA-positive farms was 3.5% (10/283) (Table S2).

### 2.2. Species Identification of MRS and MRM

The performance of the VITEK 2 and matrix-assisted laser desorption/ionization–time of flight (MALDI–TOF) VITEK mass spectrometry (MS) systems for MRS/MRM identification was assessed using WGS as the reference method. Species identification with three different methods was discordant for 10/70 isolates: 9 *M. fleuretii* isolates (misidentified by VITEK 2 and MALDI-TOF VITEK MS systems) and 1 *S. aureus* isolate (misidentified by VITEK 2 system) (Table S2). The studied 70 MRS/MRM isolates belonged to the following species according to WGS: four *Staphylococcus* species (*S. aureus* (*n* = 10), *S. epidermidis* (*n* = 10), *S. haemolyticus* (*n* = 17) and *S. saprophyticus* (*n* = 5)) and three *Mammaliicoccus* species (*M. fleurettii* (*n* = 9), *M. lentus* (*n* = 2) and *M. sciuri* (*n* = 17)). All isolates were *mecA*-positive by PCR.

### 2.3. WGS Characterization of MRS and MRM

Regarding multilocus sequence typing (MLST), *spa* typing and SCC*mec* typing, the 10 studied *S. aureus* isolates belonged to four different sequence types (STs): ST398 (six isolates; all *spa* type t034 and SCC*mec* type V), ST7882 (two isolates; t127 and t693, both SCC*mec* type IVa), ST152 (one isolate; t355 and SCC*mec* type V) and ST7 (one isolate; t091 and SCCmec type V). The basic characteristics of the constructed ad hoc (species-specific) cgMLST schemes are shown in Table S3 and the typing results for other MRS and MRM species in Table S4.

**Figure 1:**
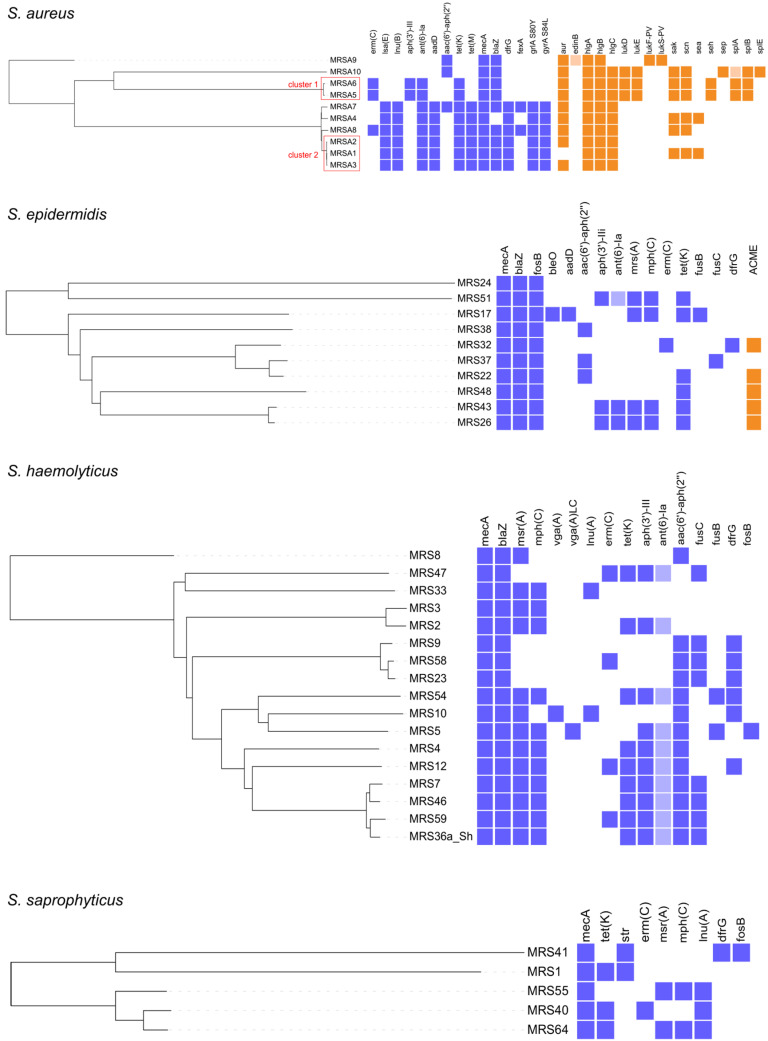
cgMLST phylogenetic tree of methicillin-resistant *Staphylococcus* spp. isolates. Violet and orange squares next to the tree denote the presence of antimicrobial resistance and virulence genes, respectively. Light colored squares represent genes with <100% identity and <100% coverage to the reference gene. Clusters of isolates differing in ≤24 cgMLST alleles are highlighted in red rectangles.

In total, 17 virulence genes were detected in MRSA isolates; each MRSA isolate harbored at least 4 of them. One isolate belonging to ST152 harbored the Panton–Valentine leucocidin (PVL)-encoding genes (Figure 1, Table S5). Other MRS and MRM species harbored no virulence genes, except for the arginine catabolic mobile element (ACME) present in 5/10 *S. epidermidis* isolates (Figure 1, Table S5). 

**Figure 2:**
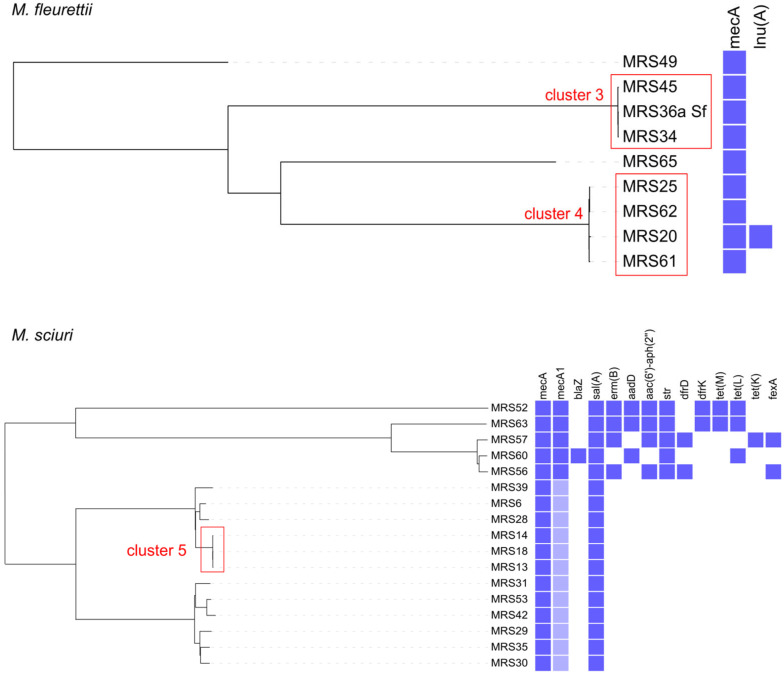
cgMLST phylogenetic tree of methicillin-resistant *Mammaliicoccus* spp. isolates. Violet squares next to the tree represent the presence of antimicrobial resistance genes. Light violet squares represent genes with <100% identity and <100% coverage to the reference gene. Clusters of isolates differing in ≤24 cgMLST alleles are highlighted in red rectangles.

In total, 31 AMR genes were detected in MRS/MRM isolates, conferring resistance to eight antimicrobial groups (aminoglycosides, beta-lactams, macrolide/lincosamide/streptogramin (MLS) group, tetracyclines, trimethoprim, phenicols, fosfomycin and fusidic acid) (Tables S6 and S7). Additionally, in *S. aureus* isolates (6/10), two chromosomal point mutations conferring fluoroquinolone resistance were identified: S80Y mutation in the *grlA*/*parC* gene and S84L mutation in the *gyrA* gene. AMR genes identified in each MRS/MRM isolate are shown in Figure 1 and Figure 2. The following AMR genes were identified in two *M. lentus* isolates: (i) *mecA*, *mph*(C), *lnu*(A), *erm*(B), *tet*(K), *fosD*, *str*, *aac*(*6*′)-*aph*(*2*″), *fexA* and *dfrG* in MRS44, and (ii) *mecA*, *mph*(C), *aac*(*6*′)-*aph*(*2*″), cat_(pC221)_ in MRS50. In total, 61.4% isolates harbored genes conferring multidrug resistance (MDR; resistance to ≥3 antimicrobial classes [36]). The MDR rate was significantly higher in MRS isolates than in MRM isolates (36/42 vs. 7/28, respectively; *p* < 0.0001, Fisher’s exact test).

Clustering based on the core genome multilocus sequence typing (cgMLST) was performed for all species with more than two representative isolates, and the constructed phylogenetic trees showed high genetic diversity within the species (Figure 1 and Figure 2). The following cgMLST clusters (≤24 cgMLST allele differences) were identified: two *S. aureus* clusters, two *M. fleurettii* clusters and one *M. sciuri* cluster (Table S4, Figure 1 and Figure 2). Clusters 1 and 2 encompassed isolates from the farms in close proximity to each other (same village; ~2 km radius), whereas other clusters encompassed isolates from geographically more distant farms (20–50 km). A single veterinarian supervised both farms associated with cluster 1. Farms associated with clusters 2 and 5 belonged to the milk-shed area of the same dairy (same raw milk supply line).

### 2.4. Risk Factors for the Occurrence of MRS/MRM in Dairy Farms

The milking system, housing system, herd size, breed, TBC and SCC were included into the logistic regression model to assess their association with the occurrence of MRS/MRM in dairy farms. The milking system and TBC were significantly associated with the occurrence of MRS/MRM in dairy farms, as assessed by the univariate risk factor analysis (Table 1), and were included in the final multivariate logistic regression model (Table 2). The Hosmer–Lemeshow test showed that the regression model was a good fit (*p* = 0.056). The regression model showed that farms with a pipeline milking system had 2.5-fold higher odds of harboring MRS/MRM than farms with a bucket milking system (*p* = 0.003). In addition, farms with TBC > 100,000 CFU (colony forming units)/mL had 2.8-fold higher odds of harboring MRS/MRM compared with farms with TBC < 100,000 CFU/mL (*p* = 0.001).

## 3. Discussion

Dairy production is the largest sector of Serbian agriculture, accounting for approximately 8% of the agriculture production value [37]. It depends on family-run farms and lacks national mastitis control programs. To this aim, we have estimated the occurrence of MRS and MRM in BTM samples from 283 bovine dairy farms located in the Belgrade district. The selected farms were representative of the Serbian dairy sector, since the vast majority (99.95%) of national agricultural holdings (AHs) specialized in milk production are family-run [38]. In addition, the analyzed farms were characterized by the tie-stall housing system and domestic Simmental dairy breed, which are also prevalent at the national level [39,40].

One quarter of the analyzed farms were positive for MRS and/or MRM, with 3.5% of farms positive for MRSA. To date, several studies have investigated MRSA prevalence in dairy farms [10,30,32,33,34,35,41,42,43,44,45,46,47,48]. In general, the prevalence of MRSA in dairy herds has been reported to be low [5,6], although an increase is expected [48]. However, differences in study designs (sample types, inoculum sizes, isolation procedures, detection methods and farm management systems) make comparisons difficult, in addition to the impact of other factors (e.g., geographic location, sanitation practices, on-farm biosecurity, antimicrobial use and mastitis control strategy) that may also influence MRSA prevalence. The low MRSA occurrence rate observed in this study is consistent with data from similar studies that used BTM cultures and a selective enrichment protocol [30,32,33,44]. Higher MRSA occurrence rates in BTM reported in other studies could be explained by pre-selection of dairy farms based on a history of MRSA-associated clinical/subclinical mastitis [42] or previous reports of MRSA from BTM [49,50].

Most previous studies on staphylococci in dairy herds have focused on MRSA, although methicillin resistance has been reported in several other *Staphylococcus* and *Mammaliicoccus* species commonly found on dairy farms [10,51,52,53,54]. Methicillin resistance rates in NAS are generally higher than in *S. aureus* [5]. In this study, non-*S*. *aureus* MRS/MRM were detected in 20.8% of BTM samples. This rate is lower than those previously reported in Switzerland (62.0%; [51]) and Germany (42.1%; [10]). Of note, in the German study, farms were selected based on previous MRSA detection. In contrast, a low incidence of non-*S. aureus* MRS/MRM in BTM has been reported in the United States (2.4%; [53]) and the United Kingdom (~5%; [54]). The low methicillin resistance rate in NAS and mammaliicocci noted in the above-mentioned studies could be due to the controlled and restrictive use of antimicrobials in dairy farming, high levels of hygiene on farms (especially during milking) and implementation of biosecurity measures [53,54]. *M. sciuri* and *S. haemolyticus* were the most frequently isolated species in this study. *M. sciuri* is widespread in nature and can be found in various animal hosts [14]. It also represents the most likely evolutionary origin of *mecA* [55]. *M. sciuri* is most commonly isolated from animal teats, whereas *S. haemolyticus* is mainly associated with milk samples [56,57] and it has a higher prevalence of the *mecA* gene compared to other staphylococci species [58]. Previous studies on the occurrence of MRS/MRM in cattle also reported a high rate of methicillin resistance in *M. sciuri* [8,50]. *M. sciuri* has also been reported as the most abundant MRS/MRM species from BTM in England and Wales [54].

In this study, TBC (>100,000 CFU/mL) and the milking system (pipeline milking) were significantly associated with the occurrence of MRS/MRM in dairy farms. Elevated TBC is an indicator of unsanitary practices on the farm, including an absence of precautionary measures for milking operation, a low milking cow cleanliness score, poor equipment hygiene and water quality, as well as high California Mastitis Test (CMT) scores and inadequate raw milk cooling [59]. Effective milking preparation and housing hygiene are paramount in preventing contagious and environmental mastitis [60]. *S. aureus* spreads from cow to cow and udder to udder, primarily during the milking process. The milking hygiene score is negatively correlated with MRSA prevalence [49]. Inadequate hygiene during milking has been observed previously in all MRSA-affected farms [50]. Biofilms in the milking equipment are also a potential source of contamination of BTM with *S. aureus* and other pathogens [61,62]. Although hygiene during milking and the presence of biofilms in the milking systems were not evaluated in this study, we assume that the lack of pre- and post-dipping protocols, improper handling and hygiene of milking unit clusters, lack of mastitis control measures such as rapid segregation and culling of infected cows are positively correlated with the MRS/MRM-positive status of the dairy farms investigated. On the contrary, the housing system, herd size, breed and SCC were not found to be significant risk factors for the MRS/MRM-positive status of dairy herds. This contrasts with previous studies that identified herd size as a risk factor for MRS/MRM occurrence, with larger herds more likely to be positive than smaller herds [32,34]. Of note, most (76.3%) of the analyzed farms were typically in the range of 1–19 cows and often kept multiple animal species including pigs, which are considered a risk factor for MRSA occurrence in dairy herds [5]. A previous study on udder pathogens identified milking and housing systems as important risk factors for the occurrence of intramammary infections and showed that these risk factors are pathogen specific [63].

MALDI-TOF MS has been successfully used to identify staphylococci from bovine milk [64,65,66]. In this study, a comparative evaluation of the VITEK 2 and MALDI-TOF VITEK MS systems in parallel with WGS as a reference method showed that nine out of ten misidentified isolates belonged to *M. fleurettii*. This is likely due to limitations of the reference databases used (VITEK 2 GP and SARAMIS), which did not include *M. fleurettii*. As previously reported [67,68], curated and updated databases are essential for optimal performance of VITEK MS automated systems. An advantage of WGS as the reference method for species identification compared with other methods is that it provides the most accurate phylogeny and the analyzed strains can be compared with any strain/species in the database, including recently described species. An additional advantage of WGS used here was the determination of AMR and virulence genes, which eliminated the need for conventional phenotypic and PCR testing.

In this study, five cgMLST clusters of MRS/MRM isolates were observed. Clusters 1 and 2 included isolates from farms located in the same village, suggesting MRSA transmission through personnel visits (e.g., farm workers, veterinarians or professional visitors). Other possible transmission vehicles include animal vectors (e.g., flies or rodents) or dust [50]. Because the farms associated with clusters 2 and 5 were part of the milk-shed area of the same dairy, milk trucks represent a likely transmission route. These trucks routinely visit multiple dairy herds on the same day, and no disinfection baths were present at the entrance to each farm. One MRSA-positive farm associated with cluster 2 (farm code 224) kept pigs in close proximity (<50 m) to dairy cows. Thus, pigs could also have been involved in MRSA transmission to dairy cows. In all cases, MRS/MRM transmission could also have occurred through local livestock trade, but such data were not available. Additional epidemiological data on the farms studied would be needed to identify the role of the above possible transmission routes in MRS/MRM transmission between herds.

Several MRSA clones have been identified in milk and dairy products worldwide, with CC398 and CC1 being the most frequently reported MLST CCs in Europe [69]. Most (6/10) MRSA isolates analyzed in this study belonged to the livestock-associated (LA) lineage ST398-SCC*mec* V-t034. Previous studies from Europe reported ST398 as the predominant clone in dairy herds [32,34,70,71,72,73]. Two MRSA isolates from our study were of CC1, one belonging to *spa* type t127. MRSA CC1 has been recognized as a successful hospital- (HA) and community-acquired (CA) MRSA lineage in humans and has also been responsible for invasive infections [74,75]. MRSA CC1-t127 has also been reported in cattle from Germany, Italy and Switzerland [34,74,76,77]. In Hungary, MRSA CC1-t127 was found in cattle with subclinical mastitis, and transmission to a farm worker in close contact with cattle was also demonstrated [78]. In this study, one of the identified MRSA isolates was of genotype ST152-t355 and harbored the PVL-encoding genes. This genotype has been linked to community cases throughout Europe and is prevalent in the Balkan region [79]. This genotype was the most common (67.5%) MRSA genotype isolated from milk and dairy products in southern Italy, and most isolates were PVL-positive [80]. It is worth noting that the ST152-t355 genotype has been reported in Serbia since 2008 [20] and it is still the dominant PVL-positive human genotype in this region [23]. Due to the zoonotic potential of MRSA [73,81], the introduction of the human-adapted MRSA genotype ST152-t355 into the analyzed farm by colonized humans cannot be excluded.

Virulence genes were distributed with different frequencies among the MRSA isolates studied, which may indicate evolution and adaptation to different niches or environments. In agreement with previous results [82], the most frequently detected virulence genes were *hlgA* and *hlgB*, followed by *aur* and *hlgC*. This is not surprising since the *aur* gene is highly conserved in *S. aureus* [83]. Most (8/10) MRSA isolates examined had the same pattern of virulence genes encoding hemolysins (*hlgA*, *hlgB* and *hlgC*) and aureolysin (*aur*) as previously described in two MRSA ST398 from BTM originating from healthy cows in China [84]. Additionally, the human immune evasion cluster (IEC) genes *scn* and *sak* were detected in three MRSA isolates of genotype ST398-t034. The re-acquisition of IEC by LA-MRSA ST398 suggests the re-adaptation of LA-MRSA ST398 to the human host and favors the (re)emergence of MRSA ST398 in humans [85,86]. Consistent with previous findings [74,87,88,89,90], both MRSA CC1 isolates examined harbored *seh*, *sak* and *scn*. Because staphylococcal enterotoxin H (SEH)-producing strains have been involved in staphylococcal food poisoning [91], the risk of such strains to cause foodborne intoxication needs to be evaluated. The presence of IEC in the MRSA CC1 isolates studied suggests that this clone may be human adapted. In addition, the CC1 isolates examined carried other protease genes (*splA*, *splB*) and *lukE/D* genes encoding leukotoxins. In addition, the two *sea*-positive MRSA ST398-t034 isolates from BTM samples could also pose a serious threat to human health. Among the classical staphylococcal enterotoxins (SE), staphylococcal enterotoxin A (SEA) is most commonly associated with staphylococcal food poisoning worldwide [92,93]. In contrast, staphylococcal enterotoxin C (SEC) is the most common staphylococcal enterotoxin produced by *S. aureus* from dairy animals [94,95], and is particularly common in strains from bovine mastitis [96], but was not detected in this study. Although the involvement of MRSA ST398 in food poisoning has not been previously reported [97], the evolving nature of MRSA ST398 and its ability to re-acquire IEC variants containing the *sea* gene [98], as observed in this study, suggest that special attention needs to be paid to the early detection of such strains.

A comprehensive WGS-based analysis of 441 bovine NAS isolates revealed a diverse repertoire of virulence genes, but a clear link between them and mastitis was not established [99]. In a recent study, the virulence profile of methicillin-resistant NAS and mammaliicocci (MRNASM) was investigated using WGS in the staphylococcal population of the nasal microbiota of dromedary camels from Algeria [100]. In agreement with the present results, among the MRNASM isolates, only *S. epidermidis* harbored virulence factors. In this study, half of the *S. epidermidis* isolates harbored ACME, which is involved in an increased ability to colonize skin and mucous membranes [101]. Consistent with this finding, nearly half of the *S. epidermidis* isolates examined from bovine mastitis milk in China carried ACME [102].

The most recent surveillance report [103] shows that 71,440 kg of antimicrobials were sold for veterinary use in Serbia in 2020, of which 218 kg were used for intramammary treatment. However, comprehensive national programs for AMR surveillance in veterinary medicine are still lacking. According to the survey conducted among Serbian livestock veterinarians, enrofloxacin was the most frequently prescribed antibiotic in mastitis therapy, followed by amoxicillin (alone or in combination with clavulanic acid), penicillin, ceftriaxone and tetracycline [19]. Although a linear relationship between antibiotic use and resistance patterns or the presence of AMR genes has not been demonstrated [104], it can be hypothesized that the frequent prescription of enrofloxacin in mastitis therapy favors the emergence of fluoroquinolone resistance, which was observed in the MRSA ST398 isolates studied. In addition to resistance to fluoroquinolones and beta-lactams, all MRSA ST398-t034 isolates examined harbored genes conferring resistance to additional antimicrobial classes (lincosamides, aminoglycosides, tetracyclines and trimethoprim). MLS resistance encoded by *erm*(C) was observed in one isolate. This is of concern since MRSA *spa* type t034 has been shown to be involved in clinical mastitis [105]. CA-MRSA clones generally have lower MDR rates and are susceptible to various non-beta-lactam antimicrobials [106,107], which is consistent with the present results where the CA-MRSA ST152-t355 isolate harbored only the *aac*(*6*′)-*aph*(*2*″) gene conferring resistance to aminoglycosides (besides the genes conferring resistance to beta-lactams).

Nobrega and colleagues [58] demonstrated that the prevalence of AMR genes in NAS from dairy herds (clinical and subclinical mastitis) was species-specific. Few studies have examined AMR genes in NAS from the milk of healthy cows [54,108]. Fosfomycin has gained attention as a combination therapy for MRSA infections. In this study, *fosB* was observed only in methicillin-resistant NAS isolates, with the highest occurrence rate observed in *S. epidermidis* (10/10). A similar occurrence of *fosB* has been reported for NAS isolated from bovine milk samples and from retail ground meat in Japan [109,110]. The MLS resistance gene *erm*(C) was identified in at least one representative of the MRS species analyzed, which contrasts with the results of a previous study in which *erm*(C) was detected exclusively in NAS species [109] or the *erm*(C) gene was not observed [108]. In this study, the lincosamide resistance gene *Inu*(A) was rarely identified in NAS isolates, namely in two *S. haemolyticus* and three *S. saprophyticus* isolates. This contrasts with the results of a previous study where *Inu*(A) was frequently identified in NAS [109]. In *S. haemolyticus*, high rates of MLS resistance genes *msr*(A) and *mph*(C) were observed, followed by *erm*(C), whereas *Inu*(A)*, vga*(A) *and vga*(A)_LC_ were less abundant. It should be noted that *mph*(C) confers high-level resistance to macrolides in *S. aureus* only when *msr*(A) is also present [111]. Contrary to the present findings, the *msr*(A) gene was not identified in *S. haemolyticus* from milk of dairy cows in China, although *mph*(C) was the most prevalent AMR gene [108]. Based on WGS-predicted susceptibility, most (36/42) MRS isolates analyzed were classified as MDR, supporting the hypothesis that MRS from cow milk are a potential reservoir for AMR genes [54,108].

The number of different AMR genes varied among *Mammaliicoccus* species: *M. fleurettii* isolates mostly harbored only *mecA*, whereas *M. sciuri* and *M. lentus* harbored AMR genes conferring resistance to six and seven antimicrobial groups, respectively. This result is consistent with previous studies [12,112]. Compared with a large number of *blaZ*-positive MRS isolates, *blaZ* was present in only one *M. sciuri* isolate (MRS60). In addition, *blaZ* was also absent in all *S. saprophyticus* isolates examined, which is in agreement with previous studies [54,109]. Although the carriage of tetracycline resistance genes is common in *M. sciuri* [12,112], *tet* genes were absent in most (12/17) *M. sciuri* isolates examined. In *M. sciuri*, three different *tet* genes were identified. *M. sciuri* isolates with multiple AMR genes conferring MDR clustered together in the cgMLST tree and were phylogenetically distant from the remaining *M. sciuri* isolates harboring only *mecA*, *mecA1* and *sal*(A). Although previously reported in *M. lentus* from dairy farms [12], the *cfr* gene, which confers resistance to a last-resort antibiotic linezolid, was not detected in the MRM isolates examined.

Two limitations of the WGS-based prediction of AMR (and other) genes used in this study should be mentioned. First, the AMR gene’s presence does not always equate to phenotypic resistance, and phenotypic susceptibility was not tested in this study. Second, the screening of AMR genes in draft genomes does not necessarily recover all AMR genes present in the genome due to the fragmented nature of the assemblies. 

## 4. Materials and Methods

### 4.1. Study Design and Sample Collection

During November 2019 and April 2021, BTM samples were collected from 283 bovine dairy farms located in the Belgrade district, covering 3234 km^2^ surface area, in the scope of quality control scheduled by the national reference laboratory for milk quality and safety. The selection criteria for participation in the study were as follows: farms that had a refrigerated bulk tank for storing and cooling of milk, cows were not milked by hand and the farmers were willing to participate in the study. The farms included in the study represented 1.4% of the total AHs specialized in milk production in Serbia. Most farms (279/283; 98.6%) were classified as family AHs. Four farms (4/283; 1.4%) were AHs with a legal entity status (AHLE). General farm data were collected through a survey that included data on farm location, herd size, breed, housing and milking system, herd average daily milk production, and SCC and TBC in BTM. Farm location data were anonymized in accordance with the general data protection regulation (farm codes 1–283). Metadata of the dairy farms under study are shown in Table S1.

The average number of cows per farm was 21 (range: 2–505). The Simmental breed was kept in 55.5% of the farms studied, whereas the Holstein-Friesian breed was in 29.3% of the farms; both breeds were kept in 15.2% of the farms. Dairy cows were kept exclusively indoors, without grazing. Tie-stall housing was the predominant housing type (92.9%), whereas on the remaining farms (7.1%) cows were kept in a loose (free-stall) system. Four types of milking systems were used: bucket (44.2%), pipeline (48.4%), milking parlor (6.4%) and automatic milking systems (1.1%) (Table S1).

The reported average daily milk yield per farm was 430 L (range: 30–13,064) (Table S1). The average SCC, expressed as a log_10_ value of somatic cells/mL (mean ± SD), was 5.40 ± 0.28 (range: 4.71–6.26). The overall average TBC, expressed as a log_10_ value of CFU/mL (mean ± SD), was 4.87 ± 0.46 (range: 3.90–6.45) (Table S1). The analyzed farms delivered raw milk to the two regional dairy plants situated in the Belgrade district. One of them collected milk from more than 14,000 farms, the other from more than 500 farms.

At each farm, a single BTM sample was collected in a sterile 50 mL container directly from the BTM cooler using a clean and sanitized dipper. Samples were immediately refrigerated and transported to the laboratory, where they were processed on the day of sampling.

### 4.2. Isolation and Confirmation of Presumptive MRS and MRM

The isolation procedure followed a two-step enrichment protocol. For primary enrichment, milk samples (25 mL) were mixed with 225 mL of Mueller–Hinton broth (Oxoid, Wesel, Germany) supplemented with 6.5% NaCl. After incubation at 37 °C for 20 h, 1 mL of the suspension was transferred to 9 mL of tryptic soy broth (Merck, Darmstadt, Germany) supplemented with 3.5 mg/L of cefoxitin (Sigma-Aldrich Chemie, Munich, Germany) and 75 mg/L of aztreonam (Sigma-Aldrich Chemie, Munich, Germany), and incubated at 37 °C for another 20 h.

After the second enrichment, one loopful (10 µL) of suspension was inoculated in parallel onto Baird-Parker (BP) agar plates (Oxoid, Basingstoke, UK) and ChromoBio MRSA chromogenic agar plates selective for MRSA (Biolab, Budapest, Hungary). Suspect MRS/MRM were collected from BP and ChromoBio MRSA agar plates, respectively. A single suspect MRS/MRM colony from each positive BTM sample was selected for further examination, except in two cases where two presumptive MRS/MRM isolates (after examining cultures for purity with subculturing on sheep blood agar plates), were recovered (farm codes 225 and 250; Table S1) and included into the analysis. All suspect MRS/MRM colonies underwent Gram staining and catalase testing.

All Gram-positive, catalase-positive colonies selected from both isolation media (*n* = 211) were screened for methicillin susceptibility by the disk diffusion test with 30 μg cefoxitin disks on Mueller–Hinton agar plates (both from Oxoid, Wesel, Germany) according to the European Committee on Antimicrobial Susceptibility Testing (EUCAST) guidelines [113]. *S. aureus* ATCC 29213 was used as a quality control strain.

For molecular confirmation of methicillin resistance, DNA was extracted from bacterial colonies by the boiling method as described previously [114]. The presence of *mecA* and *mecC* genes was tested for by a multiplex PCR according to the instructions provided by the EU reference laboratory for antimicrobial resistance [115].

### 4.3. Species Identification of MRS and MRM Using VITEK 2 and MALDI-TOF VITEK MS Systems

Species identification by VITEK 2 (bioMérieux, Marcy l’Etoile, France) was performed using GP ID cards according to the manufacturer’s instructions. The obtained results were interpreted according to the ID-GP database. *S. aureus* ATCC 29213 and *S. epidermidis* ATCC 14990 were used as quality control strains.

Species identification was also performed by MALDI-TOF MS analysis with a VITEK MS system (bioMérieux, Marcy l’Etoile, France). Before the analysis, isolates were streaked onto tryptic soy agar plates (Merck, Darmstadt, Germany) and incubated at 37 °C overnight. One colony was smeared onto target slides covered with 1 µL of VITEK MS matrix and air-dried. The acquired spectra were analyzed using the VITEK MS Plus SARAMIS Knowledge Base v4.12 database.

### 4.4. WGS and Bioinformatics Analyses

A total of 70 MRS/MRM isolates underwent WGS (Table S2). Genomic DNA was extracted using a DNA Blood & Tissue Kit (Qiagen, Hilden, Germany) according to the manufacturer’s instructions for Gram-positive bacteria. DNA libraries were prepared with the Illumina TruSeq DNA Nano Library Prep Kit (Illumina, San Diego, CA, USA). Sequencing was performed on the NextSeq 500 System using the 2 × 150 bp chemistry (Illumina, San Diego, CA, USA) to a minimum coverage of 150×. Sequencing data were submitted to the NCBI Sequence Read Archive (SRA) database under the BioProject accession number PRJNA1016076.

Raw reads were assembled with Shovill v1.18 (https://github.com/tseemann/shovill, accessed on 5 July 2023) using the --trim option and SPAdes v3.13.1 [116] as the underlying assembler with default parameters. Quality of the assemblies was assessed using Quast v5.0.2 [117]; only the genomes with *N*_50_ > 20,000 bp, number of contigs (longer than 1000 bp) less than 500 and total assembly length of ~2.8 Mbp were included into further analyses.

WGS-based species identification based on read mapping was performed using Kraken2 v2.1.2 [118], which was run with default parameters and the MiniKraken database v2. In cases of unreliable species identification using Kraken2, additional species identification based on average nucleotide identity (ANI) values was performed. To this aim, the ANI values based on the MUMmer algorithm were calculated using JSpeciesWS [119], and the 95–96% ANI threshold [120] was used for species delineation.

All MRS/MRM isolates were also screened for the presence of virulence and AMR genes. AMR genes were identified using ResFinder v4.1 [121] with default parameters. Virulence genes were identified using VirulenceFinder v2.0 (https://cge.food.dtu.dk/services/VirulenceFinder/, accessed on 11 July 2023) with default parameters and the *S. aureus* database. In addition, non-*S. aureus* draft genomes were screened for virulence genes against the Virulence factor database (VFDB; [122]) using ABRicate v1.0.1 (https://github.com/tseemann/abricate, accessed on 11 July 2023) with 60% coverage and 90% identity cut-offs. *spa* typing of *S. aureus* was performed using *spa*typer v1.0 [123]. MLST of the species with available PubMLST databases (*S. aureus*, *S. epidermidis* and *S. haemolyticus*) was performed using the Sequence query tool implemented in PubMLST [124]. SCC*mec* typing of all MRS/MRM isolates was performed using SCC*mec*Finder v1.2 [125] with default parameters. cgMLST was performed using chewBBACA v2.8.5 [126]; to maximize the discriminatory power, an ad hoc cgMLST scheme was constructed for each species with ≥2 representatives and core loci were defined as loci present in 100% genomes under study (Table S3). A neighbor-joining tree based on cgMLST allele profiles was constructed using Grapetree v1.5 [127] and was further annotated using iTol v6.7.3 [128]. A threshold of 24 cgMLST allele differences was used for cluster delineation [129].

### 4.5. Statistical Analysis

To determine the risk factors significantly associated with the occurrence of MRS/MRM in dairy farms, statistical analysis was performed using SPSS software v21 (SPSS Inc., Chicago, IL, USA). TBC and SCC thresholds were selected according to criteria set up in Regulation No 853/2004 [130]. An arbitrary value was used for herd size classification, taking into account that 87% of national AHs specialized in milk production have fewer than 10 cows [131]. For univariate logistic analysis, milking system, housing system, herd size, breed, TBC and SCC were included as independent categorical variables to identify the risk factors significantly associated with the occurrence of MRS/MRM in dairy farms (binary dependent variable). Subsequently, the variables with a significance level of *p* ≤ 0.15 in the univariate model were included in the final multivariate logistic regression model to determine the combined (adjusted) effects of the risk factors. The goodness-of-fit of the logistic regression model was assessed using the Hosmer–Lemeshow test. Results were reported as odds ratios (ORs) and their 95% confidence intervals (Cls).

## 5. Conclusions

This study provides insights into the occurrence and genomic characteristics of MRS/MRM from dairy farms in Serbia and highlights the importance of WGS to study such zoonotic opportunistic pathogens. In total, 24.0% farms were positive for MRS and/or MRM, with 70 MRS/MRM isolates identified. A high within-species genetic diversity was observed. Non-*S. aureus* MRS/MRM isolates harboring genes conferring multidrug resistance were frequently observed, highlighting their role as potential reservoirs of AMR genes. Virulence genes were rarely detected in MRS/MRM. Most MRSA isolates belonged to the typical LA-MRSA lineage ST398-t034, and one MRSA isolate of genotype ST152-t355 harbored PVL-encoding genes. Despite the low prevalence of MRSA, the presence of IEC-, SE-encoding and AMR genes in MRSA isolates from BTM poses a risk to animal and human health. Considering the zoonotic potential of MRSA, the continuous surveillance of MRSA in agriculture settings is essential. The pipeline milking system and TBC > 100,000 CFU/mL were identified as risk factors for the occurrence of MRS/MRM in BTM. These findings support the implementation of new and targeted strategies for MRS/MRM control and prevention.

## Figures and Tables

**Table 1:** Univariate risk factor analysis for the occurrence of methicillin-resistant staphylococci (MRS) and mammaliicocci (MRM) in dairy farms. Statistically significant *p* values are highlighted in bold.

Variable	*n*	MRS/MRM- Positive Farms (%)	OR	95% CI	*p*
**Milking system**					
Bucket	125	16.8	1.00		**0.008**
Pipeline	137	32.8	2.42	1.34–4.37	
Robot	3	33.3	2.48	0.26–28.57	
Milking parlor	18	5.6	0.29	0.04–2.31	
**Housing system**					
Tie-stall	263	25.1	1.00		0.146
Free-stall	20	10.0	0.33	0.07–1.47	
**Herd size (no. of dairy cows)**					
>10	194	24.2	1.00		0.908
<10	89	23.6	0.97	0.54–1.74	
**Breed**					
Simmental	157	28.0	1.00		0.216
Holstein Friesian	83	19.3	0.61	0.32–1.17	
Mix	43	18.6	0.59	0.25–1.36	
**TBC (CFU/mL)**					
<100,000	193	17.6	1.00		**0.001**
>100,000	90	37.8	2.84	1.61–4.99	
**SCC per mL**					
<400,000	227	22.5	1.00		0.218
>400,000	56	30.4	1.50	0.79–2.88	
**Total**	283	24.0			

CFU, colony forming units; CI, confidence interval; OR, odds ratio; SCC, somatic cell count; TBC, total bacterial count.

**Table 2:** Adjusted odds ratios (OR) and 95% confidence intervals (CI) for the risk factors significantly associated with the occurrence of methicillin-resistant staphylococci (MRS) and mammaliicocci (MRM) in dairy farms as assessed by multivariate logistic regression. Statistically significant *p* values are highlighted in bold.

Variable	Adjusted OR	95% CI	*p*
**Milking system**			
Bucket	1.00		
Pipeline	2.51	1.37–4.59	**0.003**
Robot	2.57	0.21–31.79	0.461
Milking parlor	0.37	0.05–3.00	0.353
**TBC (CFU/mL)**			
<100,000	1.00		
>100,000	2.78	1.55–4.97	**0.001**

CFU, colony forming units; CI, confidence interval; OR, odds ratio; TBC, total bacterial count.

## Data Availability

Raw sequencing data are available under the NCBI BioProject accession number PRJNA1016076.

## Supplementary Materials

The following supporting information can be downloaded at: https://www.mdpi.com/article/10.3390/antibiotics12101529/s1, Table S1: Farm metadata; Table S2: Isolate metadata; Table S3: cgMLST schemes; Table S4: Strain characteristics; Table S5: Presence/absence profiles of virulence genes; Table S6: Presence/absence profiles of antimicrobial resistance genes and resistance-associated mutations (staphylococci); Table S7: Presence/absence profiles of antimicrobial resistance genes (mammaliicocci).
